# Efficacy of Photodynamic Therapy in Controlling Gingival Inflammation in Orthodontic Patients: A Network Meta-Analysis

**DOI:** 10.1055/s-0045-1812493

**Published:** 2025-11-17

**Authors:** Rayssa Amaral Vieira, Carolina Martins-Pfeifer, Fabíola Galbiatti Carvalho, Eliseu Aldrighi Münchow, Márcio José da Silva Campos, Rogério Lacerda-Santos

**Affiliations:** 1 Department of Orthodontics and Pediatric Dentistry, Dental School, Federal University of Juiz de Fora, Juiz de Fora, Minas Gerais, Brazil; 2 Center for Integrative Global Oral Health, School of Dental Medicine, University of Pennsylvania, Philadelphia, PA, United States; 3 Graduate Program in Dentistry, Dental School, Federal University of Juiz de Fora, Juiz de Fora, Minas Gerais, Brazil; 4 Graduate Program in Dentistry, Dental School, Federal University of Rio Grande do Sul, Porto Alegre, Rio Grande do Sul, Brazil

**Keywords:** photodynamic therapy, orthodontics, antimicrobial agent, inflammation, gum

## Abstract

The objective of this systematic review and network meta-analysis (NMA) was to verify the effectiveness of photodynamic therapy (PDT) compared with other treatments in controlling gingival inflammation in patients undergoing treatment with fixed orthodontic appliances. An electronic search was performed in six databases and gray literature through clinical trials. The outcome of interest was the decrease in gingival inflammation, microbiological culture, and inflammatory cytokines. We conducted a random and fixed effects Bayesian NMA based on the smallest residual effect using mean difference and its credibility intervals (CI) as effect measures for the different outcomes. Six randomized clinical trials (RCTs; 173 patients) were included. It was demonstrated that treatment with PDT (MD, −0.10; 95% CI, −0.14 to −0.05) was more effective in reducing the Gingival Index compared with ultrasound (US). PDT + US treatment was superior or similar compared with US (MD, −0.36; 95% CI, −0.87 to 0.14) for decreasing *Tannerella forsythia* . In NMA, all treatments had very low certainty, demonstrating a lack of certainty regarding efficacy. The CI crossed the null effect line for treatments on the outcomes of Plaque Index, Gingival Bleeding Index, and *Porphyromonas gingivalis* ; in probing depth, this occurred for the PDT-US treatment (0.15: −0.09, 0.38), and *Fusobacterium nucleatum* , except for the PDT-US treatment (0.65: 0.0, 1.29), demonstrating very serious inaccuracy. We conclude with very low certainty that there is no strong evidence to support PDT in this treatment. The patient can benefit from either US or PDT.

## Introduction

Maintaining adequate oral hygiene represents a major challenge 1 2 3 for patients undergoing orthodontic treatment with fixed appliances. 4 5

Methods for calculus removal by the dentist, during the oral prophylaxis procedure, are performed using periodontal curettes and ultrasound (US). There are other methods that can also help with microbiological control 6 7 in the oral cavity, such as antimicrobial mouthwashes, especially those based on chlorhexidine (CHX). 8 9 However, some side effects have been demonstrated with the long-term use of CHX, such as changes in the color of teeth and tongue, changes in taste, burning sensation, and genotoxicity of oral epithelial cells. 10

At the same time, in recent years, methods such as laser irradiation, 11 and photodynamic therapy (PDT), which is associated with a photosensitizer (PS), have been increasingly used in microbiological and inflammatory control. 12 PS, when applied, accumulates intensely in the tissues. PS molecules will absorb light of the appropriate wavelength, initiating an activation process that leads cells to selective destruction. These cells present selective accumulation of PS, and with exposure to light, they are damaged by the phototoxic and oxidative reaction. 13 14 The main PSs used in dentistry are methylene blue and toluidine blue. 8 15

Although the use of diode lasers is on the rise for periodontal treatments, the difficulty in standardizing oral hygiene and individual differences brings uncertainty in the results. 11 The effects of PDT on patients using orthodontic braces have demonstrated antimicrobial effects, reducing the risk of infections, inflammation, periodontal diseases, and cavities. 5 12 14 15

Considering the importance of evaluating the use of PDT in microbiological and inflammatory control in patients during orthodontic treatment, 5 12 15 and the absence of network systematic reviews (network meta-analysis [NMA]) to analyze different treatment combinations, the objective of this systematic review and the NMA was to determine the effect of PDT on the reduction of gingival inflammation evaluated in clinical trials, measured using different indices, periodontal, microbiological culture, and inflammatory cytokines.

## Methods

### Focus Question

This systematic review was performed to answer the following clinical question: Is there any scientific evidence of the effectiveness of PDT in controlling gingival health in orthodontic patients compared with patients who received non–PDT-based treatment? PICO question and eligibility criteria are detailed in Table 1 .

**Table 1:** Criteria (PICOS, inclusion and exclusion) for study selection and research strategy

PICOS		
Participant (P)	Orthodontic patients aged at least 13 years	
Intervention (I)	Photodynamic therapy (PDT)	
Comparison (C)	Subjected to non–PDT-based treatment	
Result (O)	Primary outcome:	Decreased gingival inflammation (PD, PI, GBI, GI)
		Periodontal pocket probing depth (PD)
		Decrease in dental Plaque Index (PI)
		Decrease in the Gingival Bleeding Index (GBI)
		Decrease in the Gingival Index (GI)
	Secondary outcome:	Assessment of oral microbiological culture (OMC)
		Biochemical assessment of inflammation in gingival crevicular fluid (GCF)
Study (S)	Randomized and non-randomized clinical trials (RCT and NRCT)	
**Criteria**		
Inclusion	Articles that evaluated the influence of PDT on pathogenic microorganisms. Report the gingival and periodontal condition, minimum of 3 months of fixed orthodontic treatment in both arches, and not having received anti-inflammatory and antimicrobial therapy in the last 30 days.	
Exclusion	Patients using systemic drugs and systemic disease, have not received oral hygiene instructions and dental cleaning prior to clinical trial, use of oral rinse over the past 30 days, orthodontic retreatment, smokers, pregnant women, erupting permanent teeth, control studies without group or without microbial evaluation. Case reports, case series, animal studies, in vitro studies, editor letters, literature review and editorials.	
**Database**	**Research strategy**	
Pubmed (Medline)	((orthodontics [MeSH Terms] OR orthodontic [Title/Abstract] OR "orthodontic patients" [Title/Abstract] OR "orthodontic treatment" [Title/Abstract] OR "periodontal index" [MeSH Terms] OR "periodontal diseases" [MeSH Terms] OR periodontitis [MeSH Terms] OR periodontics [MeSH Terms] OR "periodontal patients" [Title/Abstract] OR periodontal [Title/Abstract] OR "periodontal treatment" [Title/Abstract] OR gingivitis [MeSH Terms] OR gingiva [MeSH Terms] OR "periodontitis patients" [Title/Abstract] OR caries [MeSH Terms] OR “carious dentin” [MeSH Terms] OR “caries disease” [MeSH Terms] OR “streptococcus mutans” [MeSH Terms] OR streptococcus [MeSH Terms]) AND (photochemotherapy [MeSH Terms] OR "photodynamic therapy” [Title/Abstract]) AND ("anti-infective agents" [MeSH Terms] OR antimicrobial [Title/Abstract] OR bactericidal [Title/Abstract] OR bacteriostatic [Title/Abstract] OR "anti-inflammatory agents" [MeSH Terms] OR "antiviral agents" [MeSH Teraams] OR "antifungal agents" [MeSH Terms] OR “cariogenic biofilm” [Title/Abstract] OR biofilms [MeSH Terms] OR apoptosis [MeSH Terms] OR efficacy [Title/Abstract] OR "side effects" [Title/Abstract] OR "adverse effects" [MeSH Terms] OR "collateral effects" [Title/Abstract]))	
Medline complete (EBSCO)	TX ((orthodontics OR orthodontic OR "orthodontic patients" OR "orthodontic treatment" OR "periodontal index" OR "periodontal diseases" OR periodontitis OR periodontics OR "periodontal patients" OR periodontal OR "periodontal treatment" OR gingivitis OR gingiva OR "periodontitis patients" OR caries OR “carious dentin” OR “caries disease” OR “streptococcus mutans” OR streptococcus) AND TX (photochemotherapy OR "photodynamic therapy") AND TX ("anti-infective agents" OR antimicrobial OR bactericidal OR bacteriostatic OR "anti-inflammatory agents" OR "antiviral agents" OR "antifungal agents" OR “cariogenic biofilm” OR biofilms OR apoptosis OR efficacy OR "side effects" OR "adverse effects" OR "collateral effects"))	
Web of Science (Clarivate Analytics)	TS = ((orthodontics OR orthodontic OR "orthodontic patients" OR "orthodontic treatment" OR "periodontal index" OR "periodontal diseases" OR periodontitis OR periodontics OR "periodontal patients" OR periodontal OR "periodontal treatment" OR gingivitis OR gingiva OR "periodontitis patients" OR caries OR “carious dentin” OR “caries disease” OR “streptococcus mutans” OR streptococcus) AND (photochemotherapy OR "photodynamic therapy") AND ("anti-infective agents" OR antimicrobial OR bactericidal OR bacteriostatic OR "anti-inflammatory agents" OR "antiviral agents" OR "antifungal agents" OR “cariogenic biofilm” OR biofilms OR apoptosis OR efficacy OR "side effects" OR "adverse effects" OR "collateral effects"))	
Scopus (Elsevier)	TITLE-ABS-KEY ((orthodontics OR orthodontic OR "orthodontic patients" OR "orthodontic treatment" OR "periodontal index" OR "periodontal diseases" OR periodontitis OR periodontics OR "periodontal patients" OR periodontal OR "periodontal treatment" OR gingivitis OR gingiva OR "periodontitis patients" OR caries OR “carious dentin” OR “caries disease” OR “streptococcus mutans” OR streptococcus) AND TITLE-ABS-KEY (photochemotherapy OR "photodynamic therapy") AND TITLE-ABS-KEY ("anti-infective agents" OR antimicrobial OR bactericidal OR bacteriostatic OR "anti-inflammatory agents" OR "antiviral agents" OR "antifungal agents" OR “cariogenic biofilm” OR biofilms OR apoptosis OR efficacy OR "side effects" OR "adverse effects" OR "collateral effects"))	
Cochrane (database for systematic reviews, CENTRAL, trials, protocols)	#1 orthodontics OR #2 orthodontic OR #3 "orthodontic patients" OR #4 "orthodontic treatment" OR #5 "periodontal index" OR #6 "periodontal diseases" OR #7 periodontitis OR #8 periodontics OR #9 "periodontal patients" OR #10 periodontal OR #11 "periodontal treatment" OR #12 gingivitis OR #13 gingiva OR #14 "periodontitis patients" OR #15 caries OR #16 “carious dentin” OR #17 “caries disease” OR #18 “streptococcus mutans” OR #19 streptococcus AND #20 photochemotherapy OR #21 "photodynamic therapy" AND #22 "anti-infective agents" OR #23 antimicrobial OR #24 bactericidal OR #25 bacteriostatic OR #26 "anti-inflammatory agents" OR #27 "antiviral agents" OR #28 "antifungal agents" OR #29 “cariogenic biofilm” OR #30 biofilms OR #31 apoptosis OR #32 efficacy OR #33 "side effects" OR #34 "adverse effects" OR #35 "collateral effects" #1 OR #2 OR #3 OR #4 OR #5 OR #6 OR #7 OR #8 OR #9 OR #10 OR #11 OR #12 OR #13 OR #14 OR #15 OR #16 OR #17 OR #18 OR #19 AND #20 OR #21 AND #22 OR #23 OR #24 OR #25 OR #26 OR #27 OR #28 OR #29 OR #30 OR #31 OR #32 OR #33 OR #34 OR #35	
Trials Central (http://www.isrctn.com)	Photodynamic therapy	
Clinical Trials (https://clinicaltrials.gov/)	Orthodontics *and* photodynamic therapy	

This systematic review was performed in accordance with the reference items for evaluating articles in systematic review and meta-analysis (Preferred Reporting Items for Systematic Review and Meta-Analysis [PRISMA]), 16 17 and extension statement for reports of NMAs. 18 The protocol for this NMA review has been registered on the PROSPERO platform (crd.york.ac.uk/prospero) under ID number:CRD42024504640.

### Search Strategy

An electronic search was performed in the following databases until February 3, 2025, without limitation of year or language: PubMed (Medline), Scopus, Web of Science, Medline Complete (EBSCO), Cochrane (Database for Systematic Review, CENTRAL and Protocols), and gray literature through clinical trials. The search strategies are described in Table 1 .

### Article Eligibility Criteria

Two researchers (R.A.V. and R.L.S.) independently made the selections from the abstracts, titles, and full texts, according to the eligibility criteria ( Table 1 ). Discrepancies were resolved by discussion and consensus. 19 20 21 In case of disagreements between the two evaluators and a consensus could not be reached, a third evaluator (C.M.P.) was consulted.

### Quality Assessment and Risk of Bias

Two independent reviewers assessed the risk of bias of the included studies using the Cochrane Risk of Bias for Non-Randomized Controlled Trials guidelines (ROBINS-I) tool and the Cochrane Risk of Bias for Randomized Controlled Trials (RoB2) tool. 22 23

The risk of bias for nonrandomized clinical trials (NRCTs) assessed by ROBINS-I analyzed the following seven domains: bias due to confounding; bias in the selection of study participants; bias in the classification of interventions; bias due to deviations from the intended intervention; bias due to lack of data; bias in measuring results; and bias in the selection of the reported outcome. The overall risk of bias of individual studies was classified as low (if all domains were considered to be at low risk of bias), moderate (if one or more domains were at moderate risk of bias), severe (if one or more domains were at severe risk of bias), critical (if one or more domains present a critical risk of bias).

Randomized controlled trials (RCTs) assessed by RoB2 analyzed five domains: randomization process, deviations from intended interventions, missing outcome data, outcome measurement, and selection of reported outcome) as “low risk,” “unclear risk,” or “high risk,” and disagreements were again checked by a third evaluator (C.M.P.).

### Extraction and Data Analysis

Two independent reviewers extracted the data. Disagreements were resolved through discussion until a consensus was reached. 24 The primary outcome was decreased gingival inflammation, encompassing decreased periodontal pocket probing depth (PD), dental Plaque Index (PI), Gingival Bleeding Index (GBI), and Gingival Index (GI). The secondary outcomes were the decrease in oral microbiological culture (OMC) and levels of immunoinflammatory cytokines in the gingival crevicular fluid (GCF).

### Grades of Recommendations Assessment, Development, and Evaluation

Owing to the low heterogeneity of the data and methodology of the included studies, it was possible to gather information necessary for a meta-analysis. For heterogeneous data, a narrative synthesis was approached, using a table summarizing the results according to the Grades of Recommendations Assessment, Development and Evaluation (GRADE) pro system. GRADE used the Cochrane Risk of Bias for Randomized Controlled Trials (RoB2) tool to assess the certainty of evidence for narrative synthesis. 17 25 The use of these tools was important to allow a better comparison of the evidence and the degree of certainty for recommending decision-making. 17 25

The GRADE approach assessed the number of included studies, study designs, risk of bias, inconsistency, indirect evidence, imprecision, and publication bias. Depending on the severity of the limitation in each of these categories, the evidence was downgraded by 1 or 2 levels. Based on this assessment, the certainty of the outcome assessment could be very low, low, moderate, or high quality. In NRCTs, outcomes that demonstrated a large magnitude effect were upgraded by 1 or 2 levels. For CCT, the initial certainty level was high. 17 23

### Data Synthesis and Statistical Analysis

For each continuous outcome, we collected the mean and standard deviation (SD) at baseline and each time point. We calculated the mean difference (MD) for each intervention (baseline from the last time point). The SD was calculated from the standard error (SE) obtained in the Review Manager calculator (desktop, version 5.4) for each intervention. Based on the SE, we obtained the SD for each intervention considering the following formula:  . 26

There were four treatments: US, PDT + US, PMB + US, and PDT, the latter being the reference intervention.

First, data were entered in the MetaInsight online software version 5.1.0 to run a Bayesian 27 NMA for analysis using Markov Chain Monte Carlo simulation for the following outcomes: PD, PI, GBI, *Porphyromonas gingivalis* , and *Fusobacterium nucleatum* . We considered the MD change from baseline and SD for each intervention to calculate the final effect estimate (EE): MD and respective credible intervals (95% credible intervals [CrI]) for comparing two interventions, as the studies used the same scale to measure the outcomes. We obtained each network's deviance information criterion (DIC) using fixed and random models. 27 28 The final model was chosen based on the lowest DIC ( Supplementary Material [available in the online version only]). For PD, PI, and *P. gingivalis* , we used four chains with 1:20.000 interactions. For GBI and *F. nucleatum* , there were four chains with 5.001:25.000 interactions.

Sequentially, we obtained the geometry for each outcome. Then, we assessed the convergence model based on trace plots and time-series plots. Incoherence was tested by comparing direct estimates with the indirect estimates for each comparison through the node-splitting technique. 28 The node-splitting technique did not run for PD and *F. nucleatum* due to the lack of direct and indirect evidence in a closed loop. In these cases, we checked for inconsistency throughout the global inconsistency plot. League tables were obtained for each outcome. Finally, the ranking was calculated using the surface under the cumulative ranking (SUCRA). As the ranking probability can lead to misleading conclusions, we followed the certainty of the evidence for the interpretation of data. 29

As all studies had a high or unclear risk of bias, we did not run a sensitive analysis for risk of bias. Risk of bias was considered in assessing the certainty of the evidence (please see the item below). Instead, the robustness of findings was checked by recalculating each NMA using only the last time point of each intervention to avoid inputting the SD for all interventions. Both NMAs rendered similar results. Finally, we will present here the results of the NMAs calculated using the last time point only. The network results considering the MD for the intervention are presented in the Supplementary Material (available in the online version only), as well as the results for change from baseline ( Supplementary Material [available in the online version only]).

A pairwise random effect model meta-analysis was run for the outcomes that did not have a connected network: Gingival Index and *Tannerella forsythia* . We used the final time point and SD to calculate MD and 95% CI between interventions.

Finally, three outcomes did not have at least two studies comparing the same interventions: *P. intermedia* , IL-6, and TNF-α. Therefore, we plotted the studies' SE in a series of forest plots using a fixed model. As the authors used the same scale for all outcomes, we considered MD as the final SE, except for IL-6 and TNF-α. The means and SD of these two outcomes varied on a scale from 10 to 100. In these cases, we calculated the final SE as a standardized mean difference (SMD) and 95% CI to make the SE of forest plots comparable. The Review Manager version 5.4 was used for the pairwise comparisons.

## Results

### Study Selection

**Fig. 1:**
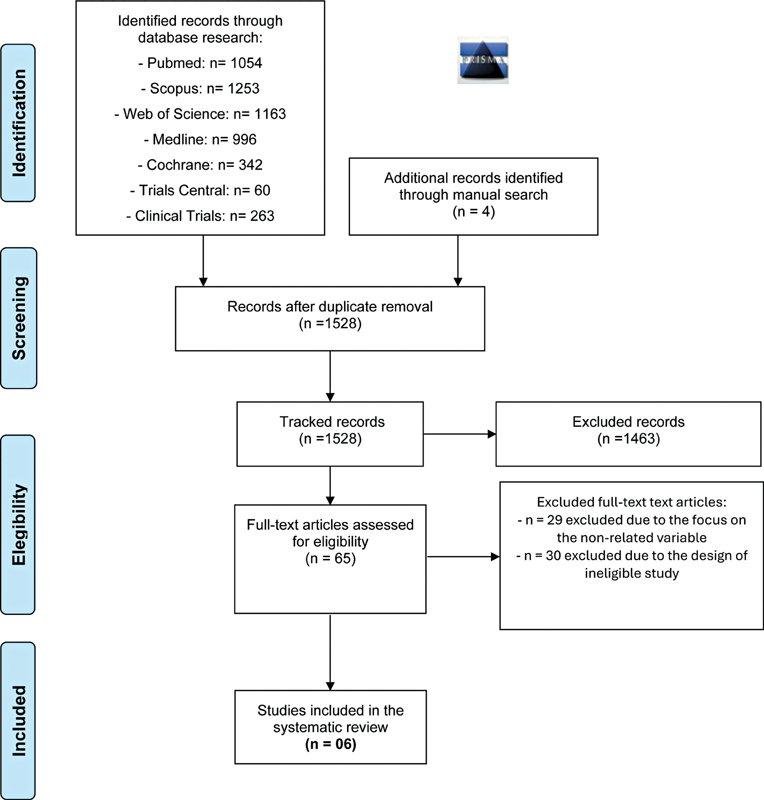
Flowchart of study selection showing the synthesis of the systematic review, in accordance with PRISMA guidelines.

After screening titles and abstracts of 1,528 articles, 65 potentially eligible articles were selected for full-text analysis, of which 6 randomized clinical trials (RCTs) 12 30 31 32 33 34 were included ( Fig. 1 ).

### Characteristics of the Studies

The studies evaluated a total of 173 patients with an average age ranging between 12 and 19 years ( Table 2 ). The studies were performed between 2015 and 2020 in Spain 12 33 and Saudi Arabia. 30 31 32 34

**Table 2:** Main characteristics of the included studies

Authors (year) Country	Study design Follow-up	No. of treated patients (P) per group	Age range (mean) Gender eligibility criteria	Treatment procedure	Conclusions	Mean difference in PD between baseline and final follow-up (mm)/ *p* -value	Mean difference in FMPI between baseline and final follow-up (%)/ *p* -value	Mean difference in FMBI between baseline and final follow-up (%)/ *p* -value	Mean difference in GI between baseline and final follow-up (%)/ *p* -value	Mean difference in OMC between baseline and final follow-up, (CFU/mL)/ *p* -value	Mean difference in GCF between baseline and final follow-up, (pg/mL)/ *p* -value
Gomez et al (2018) 33 [Spain]	RCT (full-mouth) 270 d	10P (T) 10P (C)	12–18 ♂7/♀3 (T) 15 ± 1.8y ♂7/♀3 (C) 14.2 ± 1.3y OT ≥15 m	Day 0; 15; 30; 45; 90; 180; 270 PDT 670 nm; 60 s/tooth; methylene blue 0.005% (T) US (Sonic Flex, Scaler no. 5); full-mouth (C)	Both US and PDT improved in a similar way clinical outcomes and microbiological counts during the orthodontic treatment in adolescents with fixed devices. A progression of gingival inflammation or enamel demineralization is not to be expected within 9 and 6 months, respectively, after repeated implementation of prophylactic procedures when there is sufficient oral hygiene practice	0.90 ± 0.00 (T) *p* > 0.05 0.40 ± 0.1 (C) *p* > 0.05	24 ± 4 (T) *p* > 0.05 35 ± 14 (C) *p* > 0.05	9.8 ± 2.8 (T) *p* > 0.05 6.9 ± 2.3 (C) *p* > 0.05	NR	***P. gingivalis*** 1.63 ± 0.27 (T) *p* > 0.05 0.67 ± 1.57 (C) *p* > 0.05 ***P. intermedia*** 1.30 ± 1.09 (T) *p* > 0.05 0.99 ± 0.0 (C) *p* > 0.05 ***S. mutans*** 0.09 ± 0.06 (T) *p* > 0.05 0.22 ± 0.04 (C) *p* > 0.05	NR
Abellan et al (2019) 12 [Spain]	RCT (full-mouth) 270 d	10P (T) 10P (C)	12–18 (14.6 ± 1.6) ♂14/♀6 OT ≥12 m	Day 0; 15; 30; 45; 90; 180; 270 PDT 670 nm; 60 s/tooth; methylene blue 0.005% (T) US (Sonic Flex, Scaler no. 5); full-mouth (C)	Both PDT and ultrasonic scaling are safe and effective treatment methods for gingival inflammation induced by fixed orthodontic appliances. In terms of clinical, microbiological, and anti-inflammatory outcomes, PDT was slightly more effective than the implementation of the US, allowing the extension of the benefits for a longer period	0.70 ± 0.00 (T) *p* > 0.05 0.40 ± 0.15 (C) *p* > 0.05	a 0.59 ± 0.01 (T) *p* > 0.05 a 0.59 ± 0.08 (C) *p* > 0.05 a Scale: Silness and Loe, 23 (scores: 0–3)	NR	0.90 ± 0.28(T) *p* > 0.05 0.53 ± 0.05 (C) *p* > 0.05	***P. gingivalis*** 1.63 ± 0.27 (T) *p* > 0.05 0.67 ± 1.57 (C) *p* > 0.05 ***P. intermedia*** 1.30 ± 1.09 (T) *p* > 0.05 0.99 ± 0.0 (C) *p* > 0.05 ***F. nucleatum*** 0.45 ± 0.75 (T) *p* > 0.05 0.03 ± 0.10 (C) *p* > 0.05	**IL-1β** 25.79 ± 33.95 (T) *p* > 0.05 19.05 ± 13.07 (C) *p* > 0.05 **IL-10** 5.94 ± 3.28 (T) *p* < 0.05 4.71 ± 1.76 (C) *p* < 0.05 **TNF-α** 2.27 ± 0.94 (T) *p* > 0.05 2.20 ± 0.89 (C) *p* > 0.05
Alqerban (2020) 31 [Saudi Arábia]	RCT (full-mouth) 60 d	15P (T) 15P (C1) 15P (C2)	12–19 ♂4/♀11 (T) 14.7 ± 0.8y ♂6/♀9 (C1) 16.2 ± 0.9y ♂3/♀12 (C2) 15.8 ± 0.7y OT ≥8 m	Day 0; 30; 60 PDT (670 nm, 60 s/tooth; methylene blue 0.005%) + US (T) PBM (diode laser 810 nm) + US (C1) US + manual curette; full-mouth (C2)	PDT and PBM showed similar improvement in gingival inflammatory and microbiological parameters compared to US. PDT assisted in a modest reduction of hBD-2 in patients undergoing fixed orthodontic treatment	0.78 ± 0.00 (T) *p* > 0.05 0.31 ± 0.10 (C1) *p* > 0.05 0.30 ± 0.0 (C2) *p* > 0.05	39 ± 1 (T) *p* < 0.05 39 ± 0 (C1) *p* > 0.05 31 ± 0 (C2) *p* < 0.05	12 ± 0 (T) *p* < 0.05 14 ± 0 (C1) *p* < 0.05 15 ± 2 (C2) *p* < 0.05	NR	***T. denticola*** 0.60 ± 0.60 (T) *p* < 0.05 0.42 ± 0.20 (C1) *p* > 0.05 0.37 ± 0.10 (C2) *p* > 0.05 ***F. nucleatum*** 0.23 ± 0.20 (T) *p* < 0.05 0.26 ± 0.10 (C1) *p* < 0.05 0.10 ± 0.30 (C2) *p* > 0.05	**hBD-2** 25 ± 1 (T) *p* < 0.05 17 ± 2 (C1) *p* > 0.05 6 ± 1 (C2) *p* > 0.05
Baeshen et al (2020) 32 [Saudi Arábia]	RCT (full-mouth) 28 d	15P (T) 15P (C)	14-19 ♂5/♀10(T) 16.1 ± 1.4y ♂6/♀9(C) 15.9 ± 1.3y OT ≥8 m	Day 0; 7; 28 PDT (670 nm, 120 s/tooth; methylene blue 0,005%) + US (T) US (Cavitron, Scaler); full-mouth (C)	PDT has a positive effect in significantly reducing the periodontal microbial load in established gingivitis in adolescent patients undergoing fixed orthodontic treatment.	0.60 ± 0.10(T) *p* > 0.05 0.70 ± 0.10 (C) *p* > 0.05	25.5 ± 11.9 (T) *p* < 0.05 25.3 ± 11.1 (C) *p* < 0.05	42.6 ± 16.5 (T) *p* < 0.05 30.3 ± 12.6 (C) *p* < 0.05	NR	***P. gingivalis*** 0.98 ± 0.58 (T) *p* < 0.05 0.60 ± 0.33 (C) *p* < 0.05 ***F. forsythia*** 0.83 ± 0.19 (T) *p* < 0.05 0.41 ± 0.32 (C) *p* < 0.05	**TNF-α** 19.9 ± 51.8 (T) *p* < 0.05 51 ± 25.2 (C) *p* < 0.05 **IL-6** 35.4 ± 18.0 (T) *p* < 0.05 34.8 ± 4.9 (C) *p* < 0.05
Malik and Alkadhi (2020) 34 [Saudi Arábia]	RCT (full-mouth) 180 d	18P (T) 18P (C)	16–17 ♂10/♀8(T) 16.6 ± 0.5y ♂9/♀9(C) 16.8 ± 0.4y OT ≥9 m	Day 0; 180 PDT (660 nm, 60 s/tooth; methylene blue 0,005%) (T) US (W Dental, Scaler); full-mouth (C)	The PDT is a useful adjuvant to US in reducing whole salivary oral yeast counts among adolescents undergoing orthodontic treatment. In the short-term, US with and without PDT is useful in reducing GI in adolescents undergoing orthodontic treatment	NR	NR	NR	1.8 ± 0.22 (T) *p* < 0.05 1.6 ± 0.05 (C) *p* < 0.05	**Total** 81.8 ± 5.9 (T) *p* < 0.05 24.2 ± 0.0 (C) *p* < 0.05	NR
Al Nazeh et al (2020) 30 [Saudi Arabia]	RCT (full-mouth) 28 d	11P (T) 11P (C)	16-18 (17.5y) ♂4/♀7(T) ♂5/♀6(C) OT ≥9 m	Day 0; 7; 28 PDT (670 nm, 60 s/tooth; methylene blue 0.005%) + US (T) US (Scaler); full-mouth (C)	PDT was effective in significantly reducing periodontal pathogens in established gingivitis lesions in adolescent patients undergoing fixed orthodontic treatment in the short term	NR	41.1 ± 19.8 (C) *p* < 0.05 35.4 ± 14.8 (T) *p* < 0.05	39.8 ± 23.5 (C) *p* < 0.05 48 ± 9.6 (T) *p* < 0.05	NR	***P. gingivalis*** 1.19 ± 0.62 (T) *p* < 0.05 0.26 ± 0.50 (C) *p* > 0.05 ***F. forsythia*** 1.11 ± 0.10 (T) *p* < 0.05 0.48 ± 0.35 (C) *p* > 0.05	NR

Abbreviations: C, control; FMBS, full-mouth bleeding index (%, scale, dichotomous); FMPI, full-mouth Plaque Index (%, scale, dichotomous); GCF, gingival crevicular fluid; GI, Gingival Index (%, scale, dichotomous); NR, not reported; OMC, oral microbiological culture; OT, orthodontic treatment; PBM, photobiomodulation; PD, probing depth; PDT, photodynamic therapy; RCT, randomized clinical trial; T, treatment; US, ultrasonic scaling.

a Scale: Silness and Loe 23, (scores: 0-3).

All the studies 12 30 31 32 33 34 evaluated the decrease in periodontopathogenic flora. Five RCTs 12 30 31 32 33 evaluated bag PD and full mouth PI.

Five RCTs 12 30 31 32 33 evaluated the full-mouth GBI. Three RCTs 12 31 34 evaluated the Gingival Index (GI).

Inflammatory biomarkers were evaluated in two RCTs in GCF. 12 32 In the evaluation of GCF, two RCTs 12 32 evaluated cytokines including IL-1, IL-6, IL-10, and TNF. 12 PDT in five RCTs 12 31 32 33 34 was associated with methylene blue.

As a control, three RCTs used US, 12 30 33 one RCT used US and laser, 31 and two RCTs used manual scraping. 32 34

All the studies 12 30 31 32 33 34 used the full-mouth system for treatment. And the duration of treatments varies by 4 weeks, 30 31 32 8 weeks, 31 6 months, 34 and up to 9 months. 33

### Bias Risk

**Fig. 2:**
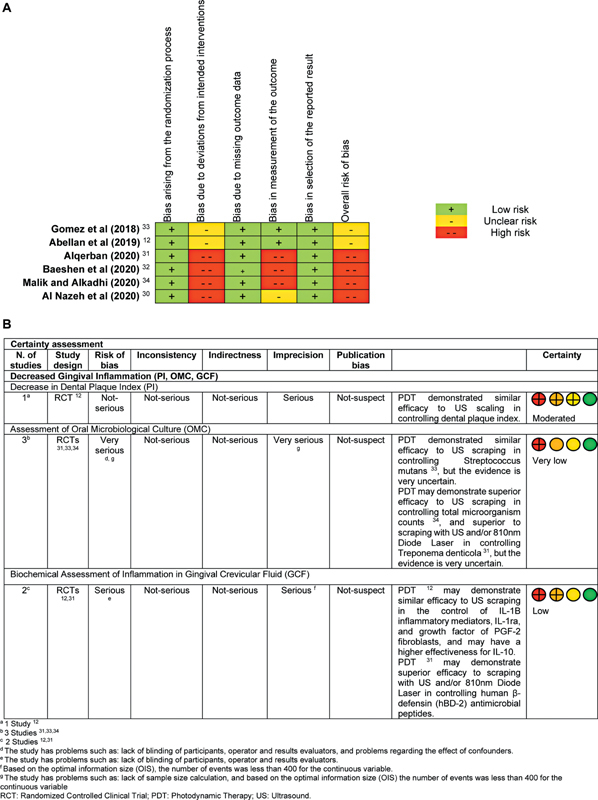
( **A** ) Risk of bias summary of included randomized trials with the Cochrane Risk of Bias Assessment Tool version 2 ( *RoB2* ). ( **B** ) Summary of findings (SoF) table according to the Grades of Recommendations, Assessment, Development, and Evaluation (GRADE) approach for narrative synthesis.

Two studies 12 33 presented an unclear risk of bias, and four studies 30 31 32 34 were identified as high risk of bias ( Fig. 2A ). In the studies 12 33 with an unclear risk of bias, the concern observed was the lack of information on deviations from the intended interventions.

Studies with high risk of bias 30 31 32 34 occurred mainly due to the lack of information on deviations from the intended interventions, such as blinding of patients and operators. Risk of bias in outcome measurement, such as lack of assessor blinding, was another concern in studies at high risk of bias.

### Results of the Included Study

#### Studies Not Eligible for Meta-Analysis

A summary in Fig. 2B describes the results and certainty of evidence using the GRADE approach for narrative synthesis of studies not included in either the paired meta-analysis or the NMA for reducing gingival inflammation on PI, OMC, and GCF outcomes.

Four RCTs 12 31 33 34 were assessed. One RCT 12 evaluated the effectiveness of PDT on PI control with moderate evidence certainty. Three RCTs 31 33 34 evaluated the effectiveness of PDT on microbiological culture control, but the evidence was very uncertain. Two RCTs 12 31 evaluated the efficacy of PDT on the control of IL-1b, IL-1ra, PGF-2, IL-10, and hBD-2, but the certainty of the evidence was low.

#### Certainty of the Evidence of the Narrative Synthesis

In the GRADE approach to narrative synthesis, the certainty of evidence was very low to moderate due to issues of risk of bias and imprecision ( Fig. 2B ).

#### Synthesis and Certainty of Evidence from Studies in Forest Graphics

Three outcomes did not have at least two studies comparing the same interventions: *Prevotella intermedia* , IL-6, and TNF-α. Therefore, we plotted the final effect estimate (EE) of the studies on a series of forest plots using a fixed model.

**Fig. 3:**
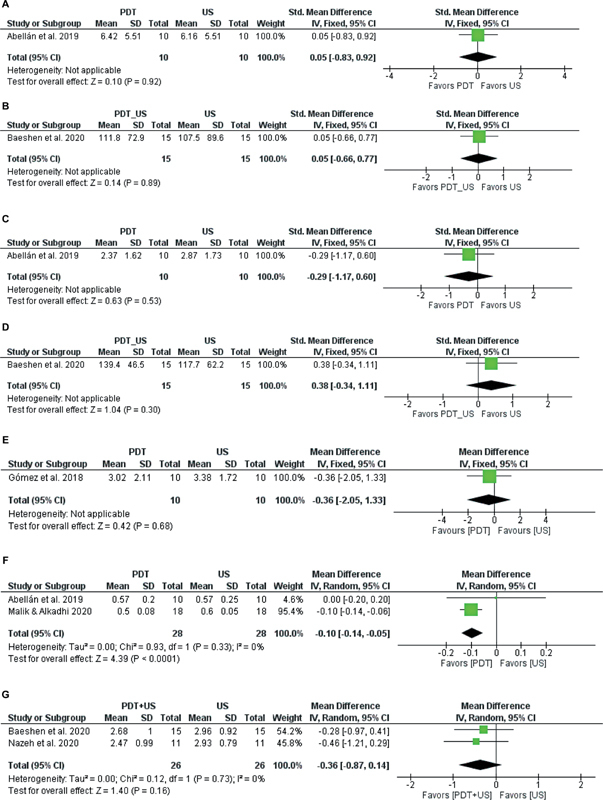
( **A** ) Forest plot showing the standardized mean difference (SMD) for IL-6 decrease between PDT therapy and US therapy. ( **B** ) Forest plot showing the standardized mean difference (SMD) for IL-6 decrease between PDT + US therapy and US therapy. ( **C** ) Forest plot showing the standardized mean difference (SMD) for decreased TNF-α between PDT therapy and US therapy. ( **D** ) Forest plot showing the standardized mean difference (SMD) for decreased TNF-α between PDT + US therapy and US therapy. ( **E** ) Forest plot showing the mean difference (MD) for *P. intermedia* decrease between PDT therapy and US therapy. ( **F** ) Meta-analysis comparing photodynamic therapy (PDT) with ultrasound (US) therapy to reduce the Gingival Index. ( **G** ) Meta-analysis comparing PDT + US therapy with US therapy to reduce *T. forythia* . ( **H** ) Network geometries. ( **A–C** ) Primary outcome, Probing Depth, Plaque Index, Gingival Bleeding Index; ( **D–E** ) Secondary outcome, *P. gingivalis* , *F. nucleatum* . ( **A** ) Probing Depth treatment network, ( **B** ) Plaque Index Treatment Network, ( **C** ) Gingival Bleeding Index treatment network, ( **D** ) treatment network for *P. gingivalis* , ( **E** ) treatment network for *F. nucleatum* . PDT, photodynamic therapy; PMB, photobiomodulation; US, ultrasonic scaling.

Fig. 3 (A, B) shows the single study SMD for IL-6. Efficacy was similar for the comparison between PDT and US (SMD, 0.05; 95% CI, −0.83 to 0.92; very low certainty; Fig. 3A , Table 3 ); and between PDT + US and US (SMD, 0.05; 95% CI, −0.66 to 0.77; very low certainty) for decreased IL-6 ( Fig. 3B , Table 3 ).

**Table 3:** Average difference (95% CI) and certainty of evidence of treatments for primary (Probing Depth, Plaque Index, Gingival Beeding Index) and secondary ( *P. gingivalis* , *F. nucleatum* ) outcomes

Probing Depth (4 trials) a			NMA	
			MD (95% CI) b	Evidence certainty
**Treatment comparison**				
**A**		**B**		
PDT	Vs.	PDT-US	−0.45 (−0.74, −0.17)	Very low c d
PDT	Vs.	PMB-US	−0.06 (−0.31, 0.19)	Very low c d
PDT	Vs.	US	0.15 (−0.09, 0.38)	Very low d e
PDT-US	Vs.	PMB-US	0.39 (0.23, 0.55)	Very low c d
PDT-US	Vs.	US	0.6 (0.44, 0.76)	Very low c d
PMB-US	Vs.	US	0.21 (0.13, 0.29)	Very low c d
**Plaque Index (5 trials)** f				
PDT	Vs.	PDT-US	−2.78 (−9.02, 3.51)	Very low c d
PDT	Vs.	PMB-US	−0.8 (−10.45, 8.73)	Very low c d
PDT	Vs.	US	−4.24 (−12.11, 3.69)	Very low d e
PDT-US	Vs.	PMB-US	1.94 (−5.65, 9.49)	Very low c d
PDT-US	Vs.	US	−1.5 (−6.96, 4.04)	Very low c d
PMB-US	Vs.	US	−3.45 (−10.6, 3.77)	Very low c d
**Gingival Bleeding Index (4 trials)** g				
PDT	Vs.	PDT-US	1.39 (−7.09, 10.32)	Very low c d
PDT	Vs.	PMB-US	3.35 (−7.05, 15.02)	Very low c d
PDT	Vs.	US	1.68 (−5.29, 10.18)	Very low d e
PDT-US	Vs.	PMB-US	1.99 (−6.15, 10.81)	Very low c d
PDT-US	Vs.	US	0.42 (−5.67, 7.18)	Very low c d
PMB-US	Vs.	US	−1.65 (−10.17, 7.08)	Very low c d
***Porphyromonas gingivalis*** **(4 trials)** h				
PDT	Vs.	PDT-US	−0.47 (−1.47, 0.53)	Very low c d
PDT	Vs.	US	0.03 (−1.17, 1.23)	Very low d e
PDT-US	Vs.	US	0.5 (−0.44, 1.44)	Very low c d
***Fusobacterium nucleatum*** **(2 trials)** i				
PDT	Vs.	PDT-US	0.57 (−0.22, 1.36)	Very low c d
PDT	Vs.	PMB-US	0.62 (−0.18, 1.41)	Very low c d
PDT	Vs.	US	0.65 (0, 1.29)	Very low d e
PDT-US	Vs.	PMB-US	0.05 (−0.38, 0.48)	Very low c d
PDT-US	Vs.	US	0.08 (−0.38, 0.54)	Very low c d
PMB-US	Vs.	US	0.03 (−0.43, 0.49)	Very low c d

Abbreviations: CI, confidence interval; DM, average difference; NMA, network meta-analysis; PDT, photodynamic therapy; PDT-US, photodynamic therapy—ultrasonic scaling; PMB-US, photobiomodulation—ultrasonic scaling; US, ultrasonic scaling.

Notes: Data are presented per NMA. Fixed effect model was used, except Gingival Bleeding Index, which used a random effect model.

None of the estimates was demoted by intransitivity.

a Four trials: Gómez et al 33 ; Abellán et al 12 ; Alqerban 31 ; Baeshen et al. 32

b Positive values favor treatment A, and negative values favor treatment B.

c Certainty in evidence downgraded by 2 levels due to very serious risk of bias.

d Certainty in evidence downgraded by 1 level due to serious risk of bias.

e Certainty in evidence downgraded by 2 levels due to very serious imprecision.

f Five trials: Gómez et al 33 ; Abellán et al 12 ; Alqerban 31 ; Baeshen et al 32 ; Al Nazeh et al. 30

g Four trials: Gómez et al 33 ; Alqerban 31 ; Baeshen et al 32 ; Al Nazeh et al. 30

h Four trials: Gómez et al 33 ; Abellán et al 12 ; Baeshen et al 32 ; Al Nazeh et al. 30

i Two trials: Abellán et al 12 ; Alqerban. 31

Fig. 3 (C, D) shows the SMD of the single study, demonstrating that orthodontic patients undergoing a comparison between PDT and US (SMD, −0.29; 95% CI, −1.17 to 0.60; very low certainty; Fig. 3C , Table 3 ); and between PDT + US and US (SMD, 0.38; 95% CI, −0.34 to 1.11; very low certainty) for decreasing TNF-α demonstrated uncertainty in the efficacy of PDT ( Fig. 3D , Table 3 ).

Fig. 3E shows the single-study MD for *P. intermedia* reduction. PDT treatment had superior or similar efficacy to US (MD, −0.36; 95% CI, −2.05 to 1.33; with very low certainty; Fig. 3E , Table 3 ).

### Meta-Analysis Results

#### Paired Meta-Analysis

A paired random effect model meta-analysis was performed for the outcomes that did not have a connected network: GI and *Tannerella forsythia* . We used the final moment and the SD to calculate MD and 95% CI between interventions.

Fig. 3 (F and G) shows the MD of paired studies for the GI. Treatment with PDT (MD, −0.10; 95% CI, −0.14 to −0.05) had a greater chance of being effective in reducing the GI compared with treatment with US (very low certainty; Fig. 3F , Table 3 ).

PDT + US was superior or similar compared with US (MD, −0.36; 95% CI, −0.87 to 0.14; very low certainty) for decreasing *T. forythia* ( Fig. 3G , Table 3 ).

#### Network Meta-Analysis

Table 3 and Fig. 3H present the “summary of findings,”, the network geometries with the four groups of treatments for the primary outcomes (PD, PI, GBI), and secondary outcomes ( *P. gingivalis* , *F. nucleatum* ). All treatments had very low certainty, which shows a lack of certainty regarding their effectiveness. Furthermore, the 95% CrI crosses the line of null effect for the treatments in the outcomes of PI, GBI, and *P. gingivalis* . For PD, this occurs with the US-PDT treatment (0.15: −0.09, 0.38). In the outcome of *F. nucleatum* , all treatments show this pattern except for the US-PDT (0.65: 0.0, 1.29), indicating very serious inaccuracy.

## Discussion

Eligible studies that demonstrated different methodologies 12 or single-study outcomes 12 31 33 34 were evaluated using the *GRADE* approach. 25 One study 12 used the numerical scale (score: 0–3) of Silness and Loe 35 for the PI, unlike the other studies 30 31 32 33 34 that employed the dichotomous scale. PDT demonstrated similar efficacy to US therapy with moderate certainty due to imprecision. In the outcome of microbiological culture, 31 33 34 for *Streptococcus mutans* , 33 PDT demonstrated similar efficacy to US; for *Treponema denticola* 31 and the total count of microorganisms, 34 PDT demonstrated superior efficacy to US 31 33 34 ; these findings do not corroborate a previous review 14 that reported the absence of significant differences between the intervention and comparison groups for *T. denticola* . However, the certainty of the evidence was very low 31 33 34 due to the risk of bias and imprecision (size of the sample).

Studies 12 31 evaluating cytokines (IL-1b, IL-1ra, PGF-2, IL-10, hBD-2) in GCF presented individual assessments without comparative analysis. PDT 12 may demonstrate similar efficacy to US therapy in controlling inflammatory mediators (IL-1b, IL-1ra). Fibroblast growth factor (PGF-2) may show superior efficacy compared to IL-10, 12 and superior to US and/or 810-nm diode laser in regulating antimicrobial peptides such as human β-defensins (hBD-2). 31 However, these findings carry low certainty due to the risk of bias (methodological problems) and imprecision related to the sample size.

Analysis of IL-6 demonstrated that PDT treatments, 8 US, 12 28 and PDT + US 32 had similar efficacy, that is, similar effects in reducing this cytokine. To reduce TNF-α, PDT 8 treatments, US, 12 28 and PDT + US 32 demonstrated uncertainty in treatments. *P. intermedia* demonstrated uncertainty in treatments. For *P. intermedia* , 33 the treatments using PDT and US had similar effectiveness, which corroborates the review findings of one study, 14 which reported no significant differences between the intervention and comparison groups.

In a previous study, 36 the authors reported that levels of microbial pathogens such as *P. intermedia* 33 as well as the inflammatory cytokines IL-6 and TNF-α 12 32 demonstrated a significant decrease in inflammatory markers after treatment with PDT compared with other treatments. Another study 14 with meta-analysis found no significant differences between treatment groups for cytokine outcomes in GCF and for bacterial analysis. These findings were not fully consistent with the present study in presenting the effectiveness of PDT treatment and the certainty of the evidence related to these outcomes and other treatments.

Bahrami et al 36 reported limitations that influence outcomes, such as variations in protocols and techniques, 12 32 which align with our findings. Previous studies 12 32 evaluating the cytokines IL-6 and TNF-α reported PDT techniques applied for 60 seconds/tooth 12 and 120 seconds/tooth. 32 Follow-up assessments were conducted at various intervals, including 0, 7, and 28 days 32 as well as 0, 15, 30, 45, 90, 180, and 270 days. 12 In this study, the meta-analysis demonstrated that PDT was more effective than the US therapy in reducing the GI, 12 34 but this evidence is uncertain. However, PDT + US and US therapy alone showed similar effectiveness in reducing *T. forythia* . 30 32 A recent study 14 did not find significant differences between the intervention and comparison groups for these outcomes. Although the present study found a higher likelihood of efficacy for PDT in improving the GI, the certainty of the evidence remains very low. 12 30 32 34

NMA results demonstrated that all treatments were similar for reducing PI, GBI, PD, *P. gingivalis* , and *F. nucleatum* . When considering the effectiveness of PDT treatment compared with other treatments, the 95% CI was wide and crossed the null effect line for the outcomes of PI, GBI, *P. gingivalis* , and PD. *F. nucleatum* presented imprecise results. The comparison between PDT and US treatment (0.65: 0.0, 1.29) favored PDT, but with very low evidence certainty, due to very serious imprecision. The findings of the present study corroborate those of Shafaee et al 14 who reported not having observed a significant difference between PDT and US treatments, or low-intensity laser therapy for reducing PI, regardless of the evaluation time.

In a previous study, 14 PDT was significantly more effective than US in the reduction of PD; however, this difference seemed to be clinically insignificant. NMA's present study showed that the data showed uncertainty in treatments, with chances of efficacy for PDT and also for US. For GBI and *P. gingivalis* , the findings of this study corroborate with study, 14 which found no significant difference for PDT compared with other treatments.

Overall, the results of this NMA showed some divergences from a review 36 and a previous MA, 14 which we credit to the different analyses performed, and to the rigor of the inclusion and exclusion criteria of the eligible studies evaluated. The previous reviews 14 36 focused on evaluating white spot lesions and gingivitis. A study without meta-analysis, 36 which restricted the criteria to only the English language and patients over 15 years old, did not report on smoking patients, pregnant women, erupting teeth, orthodontic retreatment, use of antibiotics, and anti-inflammatories. In another study with MA, 14 the authors were also not clear enough with the inclusion and exclusion criteria, specifying only the inclusion of low-intensity laser, PDT: scrapers, rinses, prophylactic treatments, and varnishes. We understand that the inclusion and exclusion criteria are important items to reduce the confounding and heterogeneity factors listed by these studies. 14 36 The MA study 14 reported that the designs of the studies by Al Nazeh et al 30 and Alqerban 31 were split-mouth, but considering the methodology of the studies and their distribution of groups, we showed that they were full-mouth assessment groups per treatment, which could impact the results in some way.

The therapeutic capacity of PDT is attributed to photodamage caused by reactive oxygen species, 14 32 36 which are cytotoxic substances that can damage the bacterial cell membrane and DNA, leading to cell death. 37 38 Studies have reported that PDT is a safe therapy. 13 39 The potential long-term oral mucosal toxicity or phototoxicity of photosensitizers on treatment is low. PDT allows frequent application, as it is a noninvasive procedure without causing cumulative toxicity. 13 39 Photocytotoxic reactions occur only in pathological tissues, in the area of distribution of the photosensitizer, allowing for narrowly selective destruction. 13 39 40 Methylene blue was the photosensitizer used in the eligible studies of this review; it has a specific cationic load that allows it to connect to gram-negative bacterial membranes, giving it high specificity to kill microorganisms. 34 40 It is not possible to say whether other photosensitizers would have more effective results in orthodontic patients.

The results of this NMA demonstrated that the patient can benefit from both US and PDT, associated or not with other treatments to control gingival inflammation, however, with low to very low certainty for the majority of the outcomes listed, which means that the estimate could change in future studies, and does not corroborate Bahrami et al 36 who reported that all investigated PDT protocols were effective. However, it is important to highlight that PDT has a high potential value in specific scenarios, such as in patients with pacemakers or other electronic cardiac devices, who should not receive US because the vibration can alter the functioning of these devices. Furthermore, patients with high tooth sensitivity, active infections, ulcerated mucosal lesions, and chlorhexidine intolerance may benefit from PDT.

The SUCRA analysis conducted in this study indicated a probability of better treatment outcomes with PDT in reducing *F. nucleatum* and GBI, with combined PDT and US in reducing PD and *P. gingivalis* , and with US alone in reducing PI. In the coherence analysis, the studies demonstrated global coherence ( Supplementary Material [available in the online version only]), and coherence between direct and indirect estimates through the knot splitting test. 41 However, with the probability presented by other therapies, it was not possible to certify the best treatment ( Supplementary Material [available in the online version only]). Moreover, the SUCRA ranking should not be considered as the final decision to make an interpretation. As SUCRA does not consider clinical thresholds and the certainty of the evidence, it can lead to misleading conclusions. 29 Instead, our study followed the certainty of the evidence to interpret data.

Regarding the comparison groups, the included studies used similar treatment protocols for PDT (i.e., 60 seconds/tooth). However, the study by Baeshen et al 32 used 120 seconds/tooth, ranging from a single irradiation session 34 to six sessions 12 33 distributed over a 9-month follow-up period. Reviews identified a distinct protocol, 14 36 highlighting considerable variability among the studies evaluated, such as application durations of 60 seconds, 14 36 180 seconds, 14 36 294 seconds, 14 and up to 10 minutes. 14 36

All the studies 12 30 31 32 33 34 of this NMA used PDT with the diode laser (light emitting diode [LED]) as a light source with a wavelength between 660 and 670 nm, different from other studies that jointly evaluated wavelengths of 450 nm 14 36 and 640 nm, 14 which may imply heterogeneity between studies and intransitivity in data from previous reviews.

All the studies 12 30 31 32 33 34 reported age symmetry. The age of the participants ranged from a minimum of 12 years old to a maximum of 19 years old, and they were not demoted due to intransitivity. Data from previous studies also mostly covered this age group. 14 36 Although this age group included only adolescents and young adults, we believe it is not possible to clearly state whether these results are applicable to adults in general, a concern not reported by other authors. 14 36

### Strengths and Limitations

The strength of this NMA lies in our rigorous methodology and well-defined inclusion/exclusion criteria to reduce the impact of heterogeneity. This is the first NMA report on PDT in orthodontic patients and its outcomes that allowed multiple treatments to be compared. In addition, the quality of evidence was evaluated by the Grade of Recommendations, Assessment, Development, and Evaluation, and all studies included were randomized. It was impossible to perform an MA comparing the treatments for some outcomes, but evaluated on the grid, due to the limited number of studies that exploit these treatments. Among the limitations, we were unable to do meta-regression for time of accompaniment due to the limited number of studies (chapter 10 of the Cochrane book) 42 —five studies were included in the NMA, and two in the pairwise meta-analysis. Another limitation was not running a subgroup analysis in NMA, which could decrease the number of studies per group, decreasing the sample size per group, and increasing the imprecision. Furthermore, the forest plots (pairwise meta-analysis) do not show statistically significant heterogeneity ( Fig. 3F : *I* ^2^ : 0%, *p* = 0.033; Fig. 3G : *I ^2^* : 0%, *p* = 0.73).

This review included a comprehensive search, incorporating five databases, two clinical trials registries, and strategies. The studies were not industry-sponsored, and the funnel plot analysis was not possible due to the small number of studies in each meta-analysis and NMA. Studies had a small sample size, and although it can be considered a source of publication bias, we did not rate down due to publication bias to avoid being penalized twice for the same reason. Instead, the certainty of the evidence was rated down due to imprecision (optimal information size not met or wide 95% CI).

We did not do a sensitivity test excluding studies at high risk of bias, due to insufficient studies, but we circumvented this problem by lowering certainty due to the risk of bias.

### Implications for Practice and Future Research

New randomized clinical trials are expected to focus on more detailed information on the implementation of randomization, allocation concealment, blinding, inclusion of larger samples, and use of validated and sufficiently clear assessment tools for different outcomes. Search for new treatment therapies or combinations of them, e.g., home brushing and toothpaste control.

## Conclusion

The hypothesis that PDT treatment is more effective in controlling gingival inflammation has not been confirmed. The null hypothesis was partially confirmed.

With very low to low certainty, there is no strong evidence to support PDT as a more effective treatment for controlling gingival inflammation in orthodontic patients. Therefore, based on the present results, the patient may benefit from either US or PDT alone or combined with other therapies. From this perspective, the most economical and simple US seems to be satisfactory.
