# Supplementary material for: Efficacy of Photodynamic Therapy in Controlling Gingival Inflammation in Orthodontic Patients: A Network Meta-Analysis
Source: Eur J Dent. 2025 Nov 17;20(2):319–33. doi: 10.1055/s-0045-1812493 (PMC13160624; doi:10.1055/s-0045-1812493)
Supplement: Supplementary file 1 — Supplementary Material [file 10-1055-s-0045-1812493-s2564336.pdf]

Supplemental Material

Global Consistency

The graph represents the contribution of each data point to the residual deviation for the NMA with consistency (horizontal axis) and the unrelated mean effect inconsistency (UME) models (vertical axis) along with the line of equality.[28] Points on the equality line mean that there is no improvement in model fit when using the inconsistency model, suggesting that there is no evidence of inconsistency.

Points above the line of equality mean they have a smaller residual deviation for the consistency model, indicating a better fit in the NMA consistency model, and points below the line of equality mean they have a better fit in the UME inconsistency model.

Residual Deviation

The leverage plot shows the average leverage across arms for each study versus the square root of the average residual deviation across arms for each study. Leverage for each data point is calculated as the posterior mean of the residual deviation, minus the deviation in the posterior mean of the fitted values. The leverage plot can be used to identify influential and/or poorly fitting studies and can be used to see how each study is affecting the overall model fit and DIC. Curves of the form $x2 + y = c$, $c = 1, 2, 3$, where x represents the square root of the residual deviation and y represents the leverage, are marked on the graph. Each point located on these parabolas contributes a quantity c to the DIC.[27,28]

Points that fall outside the line with $c = 3$ can usually be identified as contributing to poor model fit. Points with high leverage are influential, meaning they have a strong influence on the model parameters that generate their fitted values.

Probing Depth

Supplementary Table S1 Residual deviation of the NMA's randomized and fixed model (Residual Deviance—DIC), for the Probing Depth

Model fit			
**Random**		**Fixed**	
Dbar	6,538	Dbar	6,238
pD	6,497	pD	6,069
DIC	13,035	DIC	**12,308**[a]
Data points	7,000	Data points	7,000

[a]The model with the lowest DIC was used for the NMA.

Supplementary Table S2 League table de change from baseline for Probing Depth

League table[a]: fixed model			
PDT	−0.45 (−0.74, −0.17)	−0.06 (−0.31, 0.19)	0.15 (−0.09, 0.38)
0.45 (0.17, 0.74)	PDT_US	0.39 (0.23, 0.55)	0.6 (0.44, 0.76)
0.06 (−0.19, 0.31)	−0.39 (−0.55, −0.23)	PMB_US	0.21 (0.13, 0.29)
−0.15 (−0.38, 0.09)	−0.6 (−0.76, −0.44)	−0.21 (−0.29, −0.13)	US

[a]Negative values favor the treatment in the column; positive values favor treatment in the row.

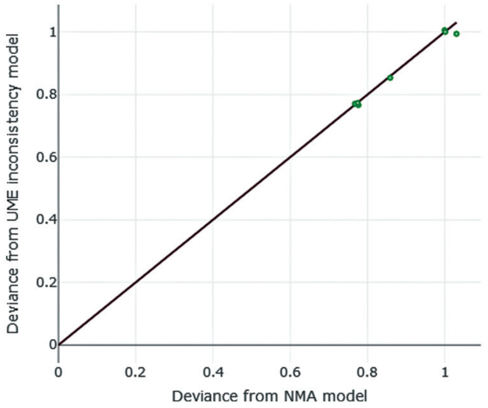

Supplementary Fig. S1 UME inconsistency model for Probing Depth.

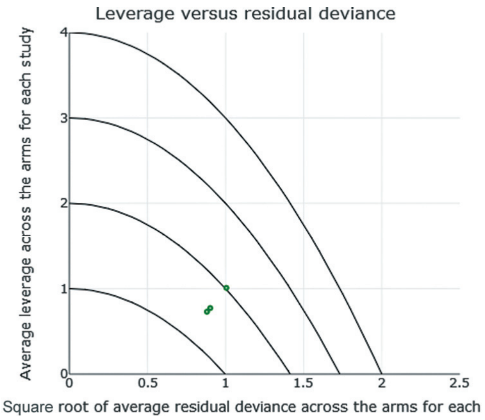

Supplementary Fig. S2 NMA model residual deviation for Probing Depth.

 © 2025. The Author(s).

Dental Plaque Index

Supplementary Table S3 Residual deviation of the NMA's randomized and fixed model (Residual Deviance—DIC) for the Dental Plaque Index

Model fit			
**Random**		**Fixed**	
Dbar	8,883	Dbar	9,380
pD	8,133	pD	7,004
DIC	17,016	DIC	**16,384**[a]
Data points	9,000	Data points	9,000

[a]The model with the lowest DIC was used for the NMA.

Supplementary Table S4 League table de change from baseline for Dental Plaque Index

League table[a]: fixed model			
PDT	−2.78 (−9.02, 3.51)	−0.8 (−10.45, 8.73)	−4.24 (−12.11, 3.69)
2.78 (−3.51, 9.02)	PDT_US	1.94 (−5.65, 9.49)	−1.5 (−6.96, 4.04)
0.8 (−8.73, 10.45)	−1.94 (−9.49, 5.65)	PMB_US	−3.45 (−10.6, 3.77)
4.24 (−3.69, 12.11)	1.5 (−4.04, 6.96)	3.45 (−3.77, 10.6)	US

[a]Negative values favor the treatment in the column; positive values favor treatment in the row.

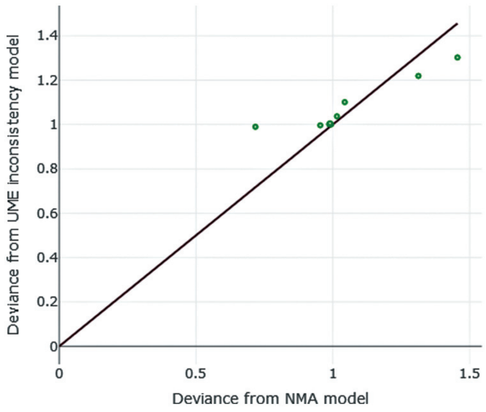

Supplementary Fig. S3 UME inconsistency model for Dental Plaque Index.

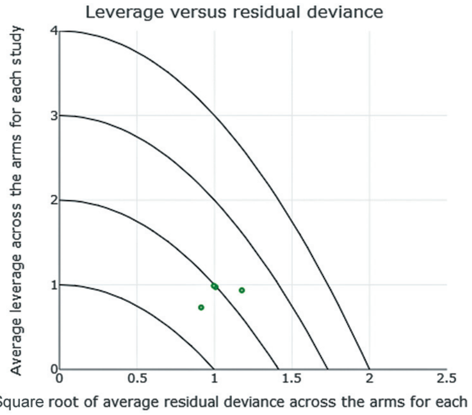

Supplementary Fig. S4 NMA model residual deviation for Dental Plaque Index.

 © 2025. The Author(s).

Gingival Bleeding Index

Supplementary Table S5 Residual deviation of the NMA randomized and fixed model (Residual Deviance—DIC) for the Gingival Bleeding Index

Model fit			
Random		Fixed	
Dbar	9,468	Dbar	12,202
pD	8,231	pD	7,012
DIC	**17,700**[a]	DIC	19,213
Data points	9,000	Data points	9,000

[a]The model with the lowest DIC was used for NMA.

Supplementary Table S6 League table de change from baseline for Gingival Bleeding Index

League table[a]: random			
PDT	1.39 (−7.09, 10.32)	3.35 (−7.05, 15.02)	1.68 (−5.29, 10.18)
−1.39 (−10.32, 7.09)	PDT_US	1.99 (−6.15, 10.81)	0.42 (−5.67, 7.18)
−3.35 (−15.02, 7.05)	−1.99 (−10.81, 6.15)	PMB_US	−1.65 (−10.17, 7.08)
−1.68 (−10.18, 5.29)	−0.42 (−7.18, 5.67)	1.65 (−7.08, 10.17)	US

[a]Negative values favor the treatment in the column; positive values favor treatment in the row.

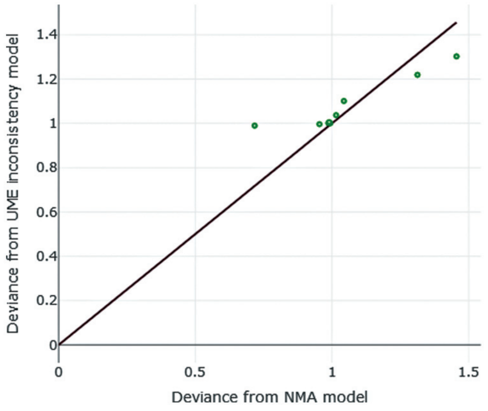

Supplementary Fig. S5 UME inconsistency model for Gingival Bleeding Index.

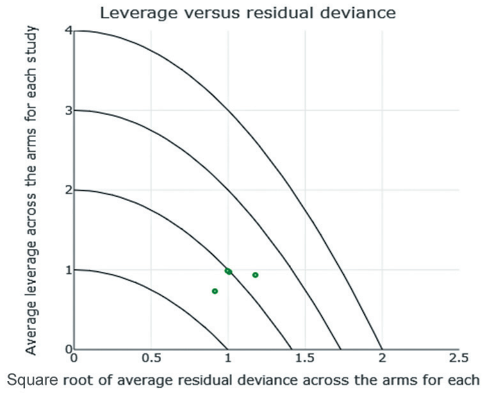

Supplementary Fig. S6 Residual deviation from the NMA model for Gingival Bleeding Index.

 © 2025. The Author(s).

P. Gingivalis

Supplementary Table S7 Residual deviation of the NMA randomized and fixed model (Residual Deviance - DIC) for P. Gingivalis

MODEL FIT			
RANDOM		FIXED	
Dbar	5,871	Dbar	6,070
pD	5,239	pD	4,998
DIC	11,111	DIC	**11,068**[a]
Data points	6,000	Data points	6,000

[a]The model with the lowest DIC was used for the NMA.

Supplementary Table S8 League table de change from baseline for *P. gingivalis*

League table[a]: fixed model		
PDT	−0.47 (−1.47, 0.53)	0.03 (−1.17, 1.23)
0.47 (−0.53, 1.47)	PDT_US	0.5 (−0.44, 1.44)
−0.03 (−1.23, 1.17)	−0.5 (−1.44, 0.44)	US

[a]Negative values favor the treatment in the column; positive values favor treatment in the row.

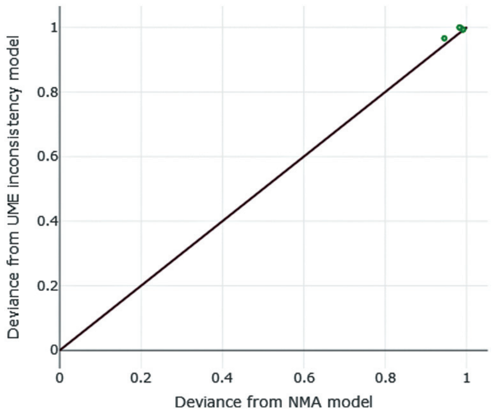

Supplementary Fig. S7 UME inconsistency model for P. Gingivalis.

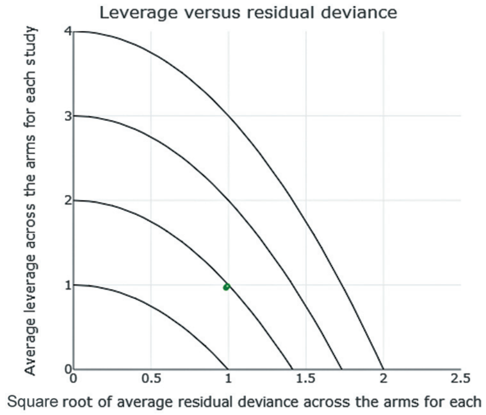

Supplementary Fig. S8 Residual deviation from the NMA model for P. Gingivalis.

 © 2025. The Author(s).

F. nucleatum

Supplementary Table S9 Residual deviation of the randomized and fixed model (Residual Deviance—DIC) of the NMA for *F. nucleatum*

Model fit			
Random		Fixed	
Dbar	5,010	Dbar	4,971
pD	5,010	pD	4,971
DIC	10,020	DIC	**9,943**[a]
Data points	5,000	Data points	5,000

[a]The model with the lowest DIC for NMA was used.

Supplementary Table S10 League table de change from baseline for *F. nucleatum*

League table[a]: fixed model			
**PDT**	**0.57 (−0.22, 1.36)**	**0.62 (−0.18, 1.41)**	**0.65 (0, 1.29)**
−0.57 (−1.36, 0.22)	PDT_US	0.05 (−0.38, 0.48)	0.08 (−0.38, 0.54)
−0.62 (−1.41, 0.18)	−0.05 (−0.48, 0.38)	PMB_US	0.03 (−0.43, 0.49)
−0.65 (−1.29, 0)	−0.08 (−0.54, 0.38)	−0.03 (−0.49, 0.43)	US

[a]Negative values favor the treatment in the column; positive values favor treatment in the row.

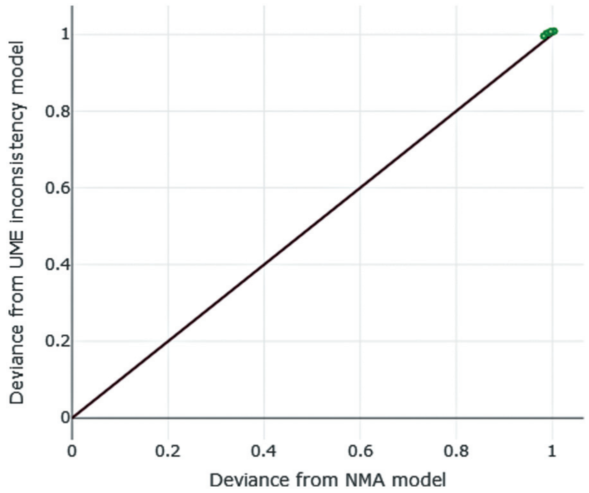

Supplementary Fig. S9 UME inconsistency model for *F. nucleatum*.

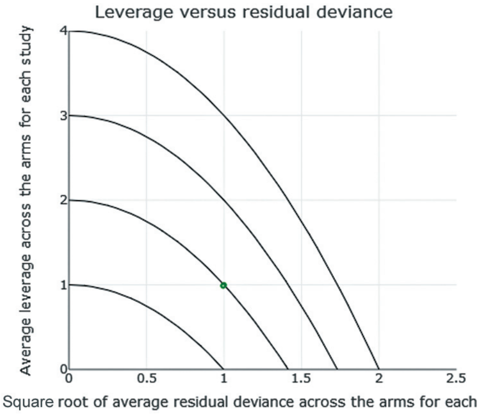

Supplementary Fig. S10 Residual deviation from the NMA model for *F. nucleatum*.

 © 2025. The Author(s).

SUCRA

Supplementary Table S11 Adjusted surface under the cumulative ranking (SUCRA) for the different treatments and outcomes

Fixed SUCRA					
Treatment	Primary outcome			Secondary outcome	
	Probing Depth	Dental Plaque Index	Gingival Bleeding Index	*P. gingivalis*	*F. nucleatum*
	%[a]	%	%	%	%
PDT	39.81	25.84	71.83	34.8	94.44
PDT + US	99.93	59.91	54.79	83.79	43.57
PMB + US	56.44	34.80	26.75	—	34.17
US	3.82	79.46	46.64	31.42	27.83

Note: Higher SUCRA indicates a greater probability of being the best treatment and 0 indicates a greater probability of being the worst.
[a]Chance of being the best treatment in percentage.

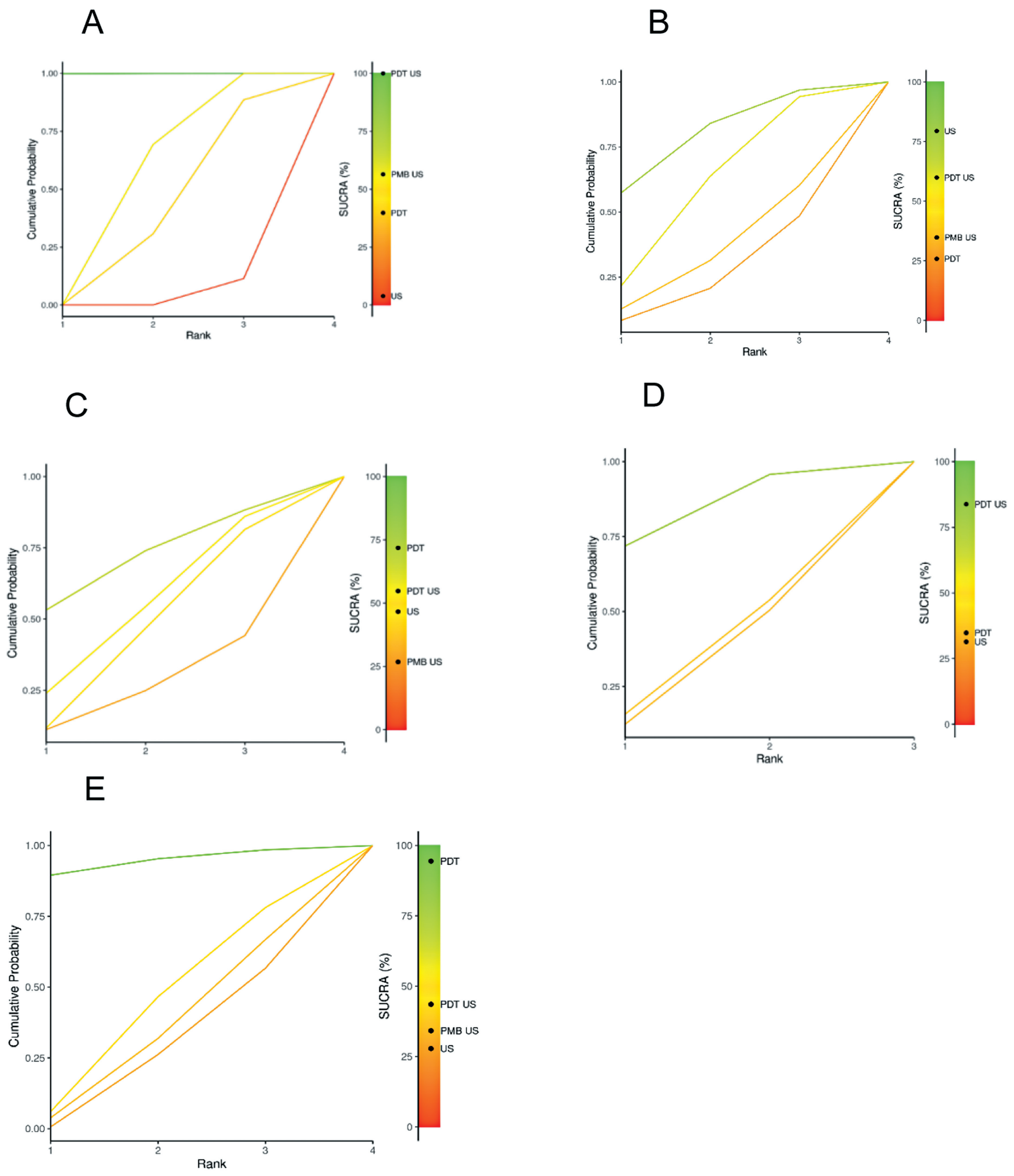

Supplementary Fig. S11 SUCRA of treatments to reduce probing depth (**A**), reduction in the Dental Plaque Index (**B**), decrease in the Gingival Bleeding Index (**C**), decrease of *P. gingivalis* (**D**), decrease of *F. nucleatum* (**E**).

 © 2025. The Author(s).
